# Unbiased and error-detecting combinatorial pooling experiments with balanced constant-weight Gray codes for consecutive positives detection

**DOI:** 10.1093/bioinformatics/btaf611

**Published:** 2025-11-13

**Authors:** Guanchen He, Vasilisa A Kovaleva, Carl Barton, Paul G Thomas, Mikhail V Pogorelyy, Hannah V Meyer, Qin Huang

**Affiliations:** School of Electronic and Information Engineering, Beihang University, Beijing 100191, China; Simons Center for Quantitative Biology, Cold Spring Harbor Laboratory, Cold Spring Harbor, NY 11724, United States; Birkbeck, University of London, London WC1E 7HX, United Kingdom; Department of Host-Microbe Interactions, St. Jude Children’s Research Hospital, Memphis, TN 38105, United States; Department of Host-Microbe Interactions, St. Jude Children’s Research Hospital, Memphis, TN 38105, United States; Simons Center for Quantitative Biology, Cold Spring Harbor Laboratory, Cold Spring Harbor, NY 11724, United States; School of Electronic and Information Engineering, Beihang University, Beijing 100191, China

## Abstract

**Motivation:**

Combinatorial pooling schemes have enabled the measurement of thousands of experiments in a small number of reactions. This efficiency is achieved by distributing the items to be measured across multiple reaction units called pools. However, current methods for the design of pooling schemes do not adequately address the need for balanced item distribution across pools, a property particularly important for biological applications.

**Results:**

Here, we introduce balanced *constant-weight Gray codes for detecting consecutive positives* (DCP-CWGCs) for the efficient construction of combinatorial pooling schemes. Balanced DCP-CWGCs ensure uniform item distribution across pools, allow for the identification of consecutive positive items such as overlapping biological sequences, and enable error detection by ensuring a constant number of tests on each item and pair of consecutive items. For the efficient construction of balanced DCP-CWGCs, we have released an open-source python package *codePUB*, with implementations of the two core algorithms: a branch-and-bound algorithm (BBA) and a recursive combination with BBA (rcBBA). Simulations using *codePUB* show that our algorithms can construct long, balanced DCP-CWGCs that allow for error detection in tractable runtime.

**Availability and implementation:**

The source code of codePUB is available at https://github.com/meyer-lab-cshl/codepub, with detailed documentation at https://codepub.readthedocs.io/.

## 1 Introduction

**Figure 1.**
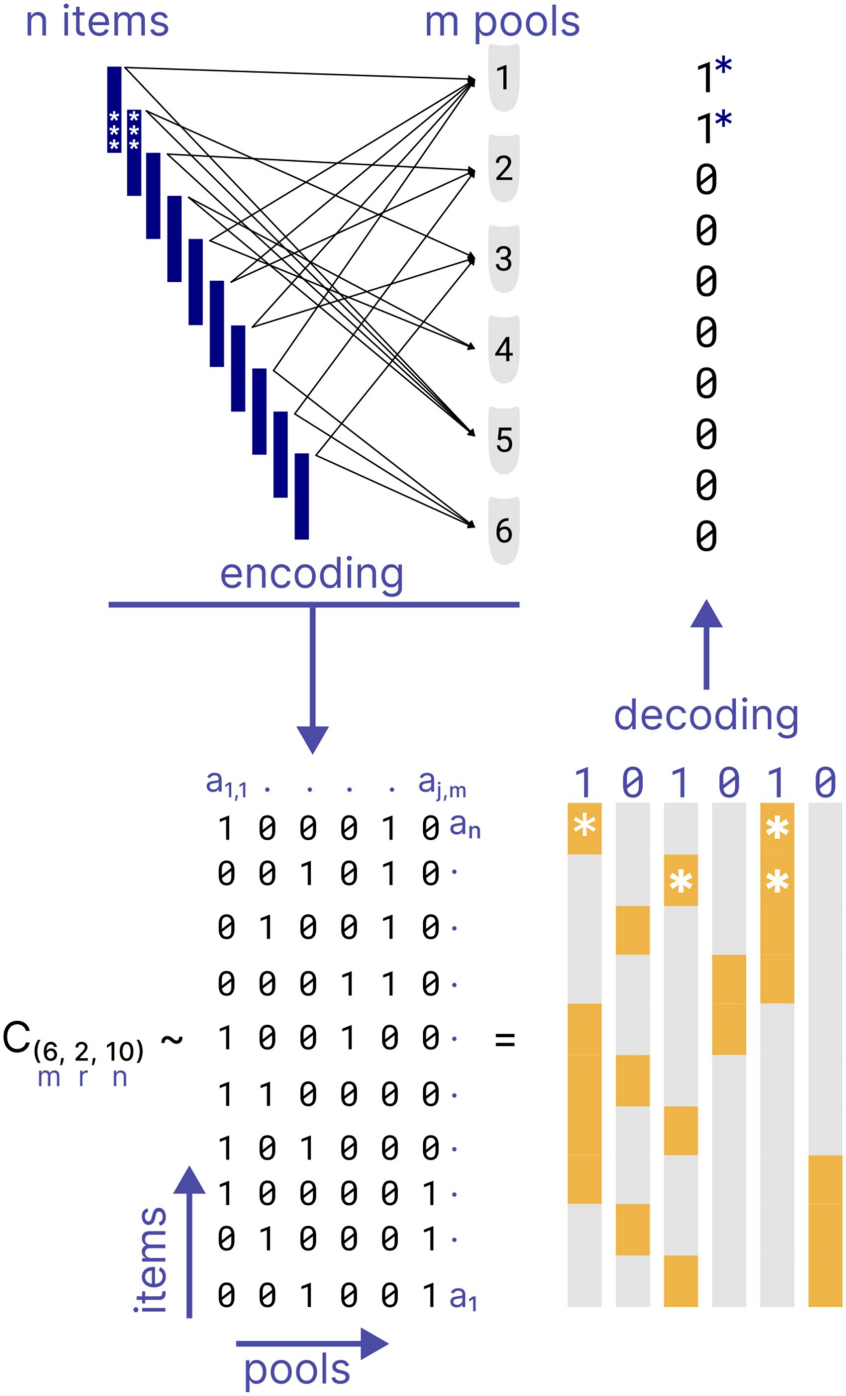
A combinatorial pooling example with 10 items and 6 pools. Items are mixed into pools according to an encoding based on a DCP-CWGC and color-coded by the expected experimental outcome (grey-negative, yellow-positive). Knowing the DCP-CWGC, the experimental outcome can be decoded and the items of interest, as indicated by stars, identified.

High-throughput experiments have become a staple across many domains of biomedical research. In these experiments, the aim is to obtain measurements for hundreds or thousands of samples in a single experimental setup. To reduce the experimental complexity and associated cost, combinatorial pooling strategies have been developed. In general terms, a combinatorial pooling experiment uses an encoding scheme according to which items (samples) are mixed such that each item is included across multiple pools and each pool contains multiple items. The experimental read-out happens on the pool level, where the measurements across pools yield a unique pattern of signals. This pattern of signals is then decoded to identify the individual-level item measurements (Fig. 1).

Since the number of pools is typically much smaller than the total number of items, combinatorial pooling offers better efficiency than individual testing. In addition, well-designed encoding/decoding schemes will allow for experimental error detection by taking advantage of items being present across multiple pools.

**Figure 2.**
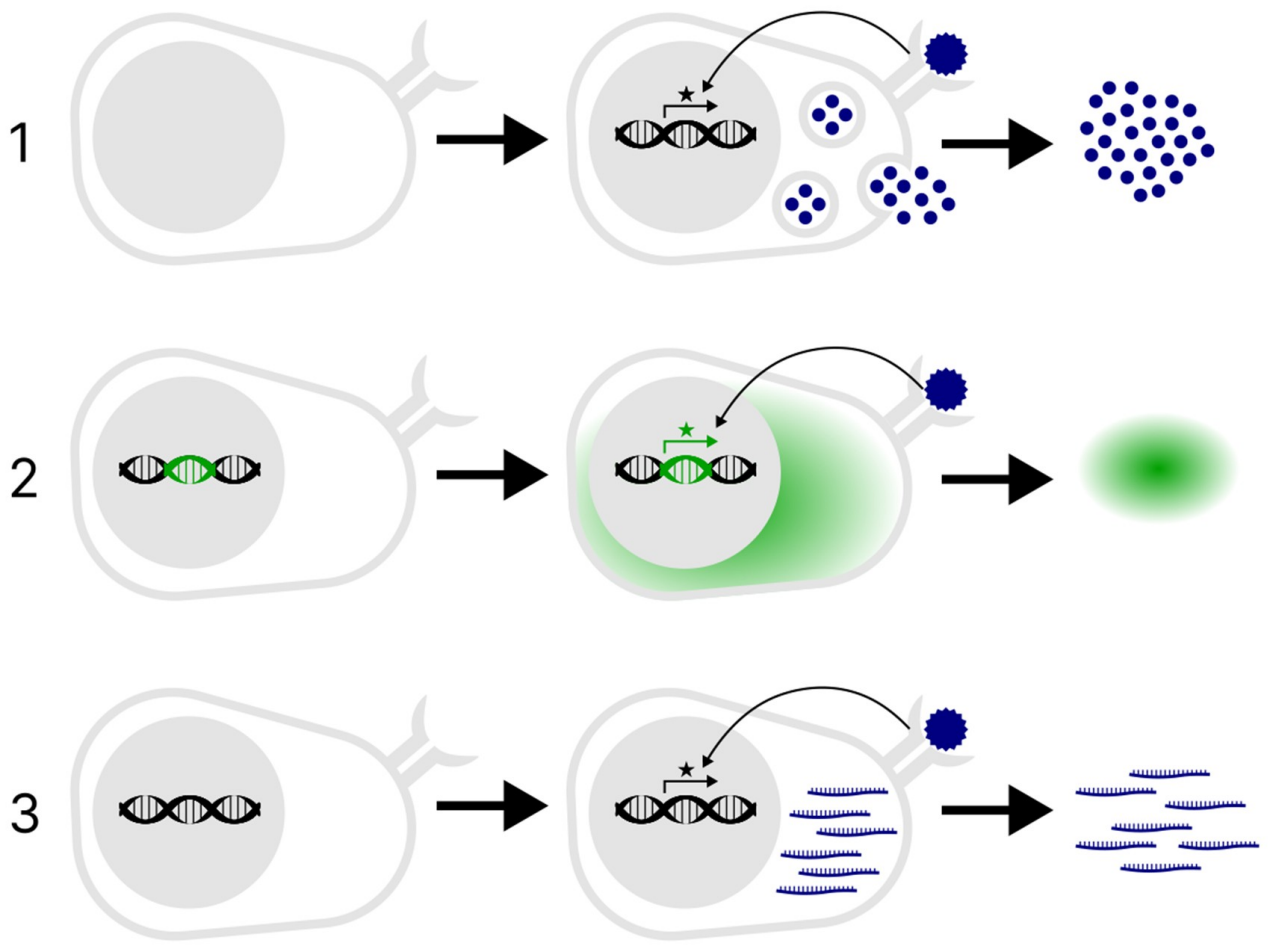
Experimental assays used in combinatorial pooling experiments. In each example, a response can be observed upon successful binding of the item (blue circle) to a cell surface receptor. The item(s) that yield such a response are considered positive. Examples of responses and their read-outs are: 1. A primary cell line which secretes activation markers such as cytokines, quantifiable by biochemical assays, 2. a reporter cell line, which produces a fluorescently labeled protein quantifiable by e.g. flow cytometry or microscopy, 3. gene expression changes in primary cells, quantifiable by RNA-sequencing.

**Figure 3.**
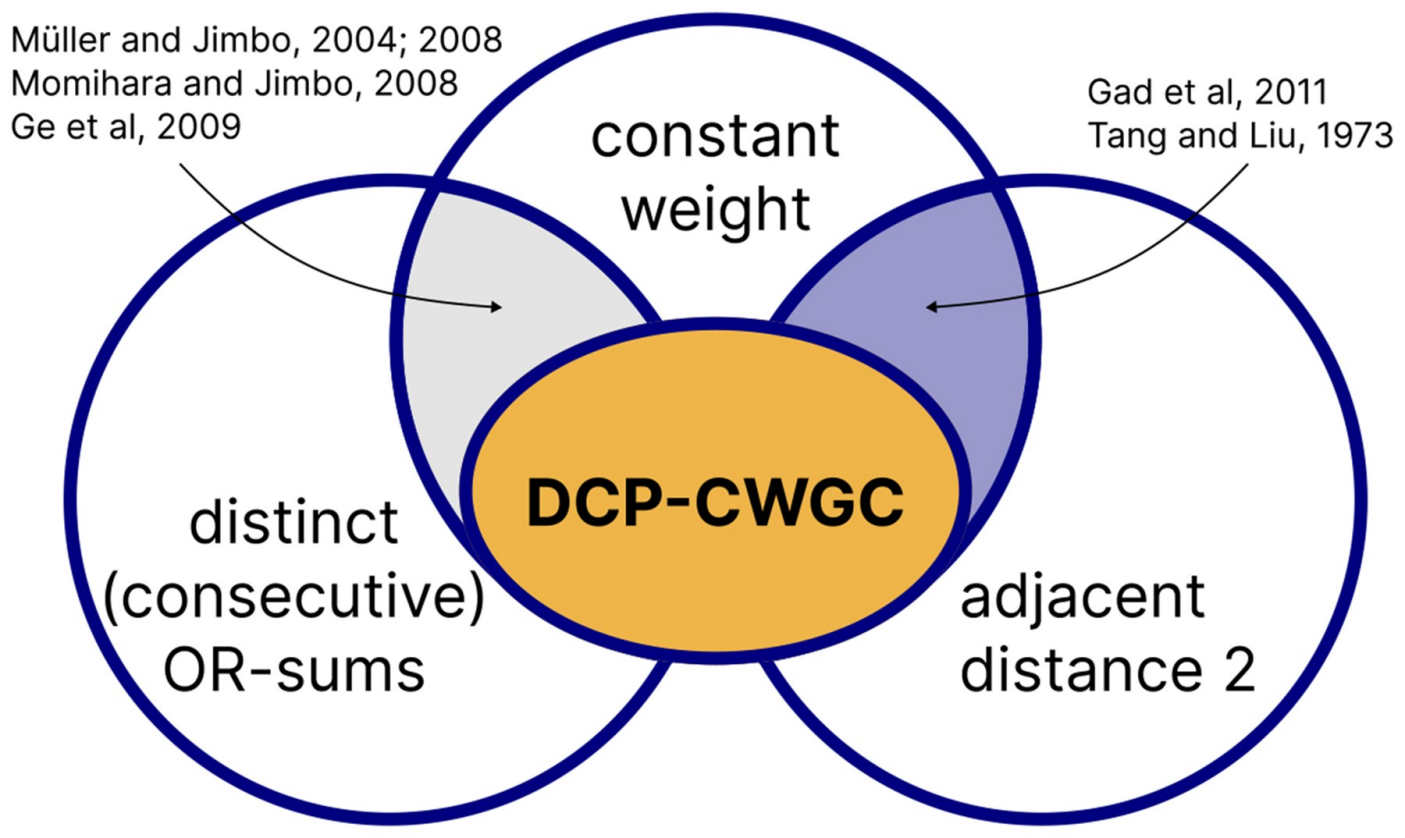
Properties of existing coding schemes. DCP-CWGCs bridge the gap between existing codes capable of consecutive positives detection (grey) and with constant weight of both codewords and consecutive OR-sums (blue).

For biological applications, the total number of pools, items per pool and the number of tests per item are key experimental parameters to consider. Minimizing both the number of pools and items per pool is crucial in biological assays with limited input material, for instance when assessing the response of patient-derived, primary cells to stimuli (items) by measuring the secretion of activation markers (Fig. 2-1). In cases where the input material is less constrained, such as using a reporter cell line (Fig. 2-2), maintaining a constant number of tests per item is crucial to prevent measuring bias in the experiment. All these factors intersect in experiments where experimental conditions and read-out are constrained by volume and range, such as measuring gene expression in a heterogeneous cell population using RNA sequencing (Fig. 2-3). Therefore, the experimental design should enable adjusting parameters to minimize the number of pools and items per pool, while also ensuring a constant number of tests per item for balanced testing. Designing a pooling scheme that adheres to these constraints and allows for error detection is non-trivial. This is further compounded when the items to be tested are non-independent, a common case in biological pooling experiments. For instance, in schemes designed to detect positive items within a pool of protein sequences, sequences are often overlapping, and these consecutive sequences will yield non-independent positive signals.

Here, we propose balanced *constant-weight Gray codes for detecting consecutive positives* (DCP-CWGCs) as a new combinatorial pooling scheme addressing these experimental considerations. Our scheme organizes items into pools following a structured sequence called DCP-CWGC, which consists of distinct binary vectors (binary addresses of items) indicating specific pools into which items are mixed. Binary addresses have a constant Hamming weight, while adjacent addresses have a Hamming distance 2 and unique OR-sums. These DCP-CWGC-defining constraints enable the identification of any consecutive positive items, keep the number of tests on each item and on each pair of consecutive items constant, and facilitate error detection. To ensure stable and unbiased detection results across pools, particular attention is placed on DCP-CWGCs that produce *balanced* pooling arrangements, where the number of items per pool is approximately constant across all pools.

To construct balanced DCP-CWGCs with flexible parameters, we developed a *branch-and-bound algorithm* (BBA), which efficiently constructs DCP-CWGCs with near-optimal balance for codes with short and moderate lengths (3000 items in less than 250 seconds). BBA conducts a heuristic depth-first search for a balance-optimized path in the bipartite graph formed from the addresses of items and the unions of consecutive addresses. Furthermore, we show that a long code can be constructed by a recursive combination of several short codes. We prove that for a range of parameter combinations, the recursive approach achieves maximal lengths and perfect balance. To broaden the applicability outside this parameter range, we implemented an extension of BBA that constructs long codes by recursive combination of several short, BBA-generated codes. This *recursive combination together with branch-and-bound algorithm* (rcBBA) efficiently extends achievable code lengths, while maintaining a near-perfect balance. BBA and rcBBA are both implemented in an open-source software *codePUB*, and simulation results show that they construct balanced DCP-CWGCs for thousands of items in tractable time.

Our balanced DCP-CWGC scheme will find applications across many branches of biological research. Its ability to ensure a constant number of tests on consecutive items makes it particularly useful for protein and DNA pooling experiments, where consecutive items represent overlapping amino acid or nucleotide sequences. For instance, a common effort in adaptive immunology research is identifying the epitopes that T cell and B cell receptors are reactive against. These epitopes are short peptide sequences generated from cellular proteins and their identification out of the large pool of possible peptides can be aided by combinatorial pooling experiments (Sospedra *et al.* 2003, Klinger *et al.* 2015, Nolan *et al.* 2025), where DCP-CWGC-based experimental designs can greatly reduce the experimental complexity. In plants, CLE peptides are critical signaling molecules for cell division and differentiation. These peptides are generated from long precursor proteins and a DCP-CWGC-based combinatorial pooling experiment could aid in systematic screens to locate mature CLE peptides within these precursors (Gao and Guo 2012). New methods developed for single-cell genomics have enabled large-scale genetic screening studies, such as the genome-wide identification of genes and their regulatory regions (Gasperini *et al.* 2019, Alda-Catalinas *et al.* 2024). While these approaches have already implemented highly multiplexed screening methods, they did not comprehensively assess the regulatory DNA sequences surrounding each gene. DCP-CWGC-based combinatorial pooling experiments could facilitate a comprehensive survey by densely tiling candidate DNA regions of interest.

In the remainder of this paper we will introduce prior work on combinatorial pooling designs and combinatorial Gray codes for consecutive positives detection (Section Preliminaries); define DCP-CWGCs and balanced DCP-CWGCs (Section Balanced Constant-Weight Gray Codes for Detecting Consecutive Positives); describe BBA and the recursive combination approach (Sections 4 and 5, respectively); and, lastly, present the computational complexity analysis and simulation results of BBA and rcBBA (Section Computational Complexity and Simulation Results).

## 2 Preliminaries

### 2.1 Combinatorial pooling designs

Consider there are *n* items $X=\{x_{1},x_{2},\ldots ,x_{n}\}$ and *m* pools $Q=\{q_{1},q_{2},\ldots ,q_{m}\}$. A combinatorial pooling design is characterized by a code comprising *n* binary vectors, $C=\{a_{1},a_{2},\ldots ,a_{n}\}$, where each vector $a_{j}=(a_{j,1},a_{j,2},\ldots ,a_{j,m})$, j=1,…,n, is called the (binary) address of the item $x_{j}$. For 1≤j≤n,1≤i≤m, $a_{j,i}=1$ indicates that the *j*-th item $x_{j}$ is contained in the *i*-th pool $q_{i}$; $a_{j,i}=0$ otherwise. For the unique interpretation of each item, all addresses in the code should be different. This code is identical to an m×n incidence matrix $H={\left(a_{j,i}\right)}_{1\leq i\leq m,1\leq j\leq n}$ over a binary Galois field GF(2), where each column in *H* is the transpose of a binary address in the code (Fig. 1, lower panel). The columns of *H* correspond to the items, and the rows of *H* correspond to the pools. As long as there is no ambiguity, hereafter we use the concept of code for a combinatorial pooling design and its corresponding incidence matrix interchangeably.

Combinatorial pooling design requirements differ from application to application and have thus motivated the construction of codes with specific properties, such as disjunctness and separability. For a comprehensive survey, the reader is referred to Du and Hwang (2006, 2000). Here, we will focus on a special class that takes the shape of combinatorial Gray codes (Savage 1997, Mütze 2024) for consecutive positives detection, where each binary address differs from its consecutive ones by a “small” change.

### 2.2 Combinatorial Gray codes for consecutive positives detection

Colbourn introduced combinatorial Gray codes for the design of combinatorial pooling experiments with consecutive positives detection (Colbourn 1999). He considered a general case where *n* items to be tested are linearly ordered and positive items are within a consecutive set of size at most *d*. He illustrated that by uniformly partitioning the *n* linearly ordered items into $\left\lceil \frac{n}{d-1}\right\rceil$ groups, the general case of detecting d≥2 consecutive positive items can be simplified to the case of d=2. Since then, a number of code designs specifically address error detection and correction for combinatorial pooling experiments with consecutive positive detection. While different in construction, these codes are all equivalent to sequences comprising distinct binary addresses. In the first of a series of papers, Muller and Jimbo introduced m×n 2-consecutive positive detectable matrices with fixed constant column weight *r* (Müller and Jimbo 2004). They proved the existence of maximal 2-consecutive positive detectable matrices with $n=\left(\begin{array}{c} m\\ r \end{array}\right)$ for any m∈N and $r,1\leq r\leq \left\lfloor \frac{m}{2}\right\rfloor$. In follow up work (Müller and Jimbo 2008), they recursively construct cyclic sequences of all *r*-subsets of {1,2,…,m} with distinct consecutive unions, in which these unions are either all of even order or all of odd order. These sequences enable the detection of up to 1 error, and are proven to exist for r=2,3,…,7 and sufficiently large *n*. Orthogonal construction approaches use block sequences of maximal *t*-packings, where all blocks and all unions of two consecutive blocks consist of an error correcting code with minimum distance 4. Such sequences can not only identify any 2 consecutive positives but also correct a single error (Momihara and Jimbo 2008, Ge *et al.* 2009).

All codes above (Müller and Jimbo 2004, 2008, Momihara and Jimbo 2008, Ge *et al.* 2009) offer constant weight and distinct OR-sums, which ensure the unique identification of each pair of consecutive items (Fig. 3, grey). However, they are limited in the parameter range for which codes can be generated and cannot ensure balance property when shortened to flexible lengths. Moreover, in these codes, the OR-sums of consecutive addresses are not necessarily constant-weight, thus they do not necessarily provide an equal number of tests for each pair of consecutive items. Another set of codes, originally developed for local rank modulation in storage systems and A/D conversion in communication systems (Gad *et al.* 2011, Tang and Liu 1973), offer constant-weight and constant distance between adjacent codewords (Fig. 3, blue), yielding constant weight for OR-sums of consecutive codewords. However, they do not fulfill the OR-sums constraint and thereby cannot be applied to the consecutive positives detection. The synthesis of properties of these codes characterizes our proposed DCP-CWGCs.

## 3 Balanced constant-weight Gray codes for detecting consecutive positives

In this section, we first define DCP-CWGCs based on their key features. We then continue with a characterization of their balance property which ensures a consistent number of items across pools and therefore stable detection results across pools in biological combinatorial pooling designs.

### 3.1 Definition of DCP-CWGCs

Definition 1. An (m,r,n) DCP-CWGC is a sequence of distinct binary addresses $C=\{a_{1},a_{2},\ldots ,a_{n}\}$, $a_{j}=(a_{j,1},a_{j,2},\ldots ,a_{j,m}),j=1,\ldots ,n$, such that the following three constraints are satisfied:

1. Distinct OR-sums constraint: ∀j,k∈{1,2,…,n−1},j≠k,
  (1) $$a_{j}\vee a_{j+1}\neq a_{k}\vee a_{k+1},$$
  where ” ∨ ” represents the bitwise OR-sum of two binary vectors.
2. Constant-weight constraint: ∀j∈{1,2,…,n},
  (2) $$wt(a_{j})=\sum_{i=1}^{m}a_{j,i}=r.$$
3. Adjacent distance constraint: ∀j∈{1,2,…,n−1},
  (3) $$D_{H}(a_{j},a_{j+1})=2,$$

where “ $D_{H}$ ” represents the Hamming distance of two binary vectors.

An (m,r,n) DCP-CWGC directly corresponds to a pooling design with *m* pools, the address weight *r*, and *n* items. Constraint (1) ensures the unique identifier for each pair of consecutive items. Constraint (2) ensures the constant number of tests for each item. To ensure consistent experimental results for consecutive positives detection in a pooling scheme, an equal number of tests for each pair of consecutive items is needed. This means that the weight $wt(a_{j}\vee a_{j+1})$ should be a constant, since each pair of consecutive items is tested in the pools indicated by the OR-sum of the corresponding addresses $a_{j}\vee a_{j+1}$. Thus, we propose the adjacent distance constraint (3) for consecutive codewords, $D_{H}(a_{j},a_{j+1})=2$. With $wt(a_{j})=wt(a_{j+1})=r$, this adjacent distance constraint ensures that $a_{j}$ and $a_{j+1}$ differ in exactly one pool, which yields $wt(a_{j}\vee a_{j+1})=r+1$ and leads to a constant number of tests for each pair of consecutive items. Therefore, as long as we know whether there is a single positive item or a pair of consecutive positive items, we can always detect at least 1 error based on the number of positive pools in the experiment.

Throughout the paper, we use the following notation. We denote the ensemble of all (m,r,n) DCP-CWGCs by DCP-CWGC(m,r,n). The ensemble of all DCP-CWGCs with parameters (m,r) and arbitrary length is denoted by DCP-CWGC(m,r). We denote the set of all binary vectors of length *m* and weight *r* by S(m,r). An address *a* can be denoted as its binary form, e.g. a=(1,1,0,0,0,1), or as a corresponding index set I(a)={1,2,6}. Consequently, the bitwise OR-sum of two binary addresses is equivalent to the union of their index sets. Hereafter, as long as there is no ambiguity, we use “unions of index sets of addresses”, “unions of addresses” and “bitwise OR-sums of addresses” interchangeably.

The next proposition gives an upper bound of *n* with respect to *m* and *r* for a DCP-CWGC.

Proposition 1. The length n of any code in DCP-CWGC(m,r) is upper bounded by

(4) $$n\leq \min\left\{\left(\begin{array}{c} m\\ r \end{array}\right),\left(\begin{array}{c} m\\ r+1 \end{array}\right)+1\right\}.$$

Proof. Consider a code $C=\{a_{1},a_{2},\ldots ,a_{n}\}$ in the ensemble DCP-CWGC(m,r). The addresses in *C* all come from the set S(m,r), and they are pairwise distinct. Thus, $n\leq \left(\begin{array}{c} m\\ r \end{array}\right)$. The OR-sums of consecutive addresses in *C* all come from the set S(m,r+1), and they are pairwise distinct. Thus, $n-1\leq \left(\begin{array}{c} m\\ r+1 \end{array}\right)$. □

### 3.2 Balanced DCP-CWGCs

For balanced DCP-CWGCs, the sum of each row in their corresponding incidence matrices should be approximately constant. To characterize the balance property of a code C∈DCP-CWGC(m,r,n), we define its balance vector as $W_{C}=(w_{1},w_{2},\ldots ,w_{m})$, where


$$w_{i}=\sum_{j=1}^{n}a_{j,i},1\leq i\leq m.$$

The deviation of the balance vector, $\delta_{C}:=\max(W_{C})-\min(W_{C})$, is a critical evaluation criterion of the balance property of *C*. The smaller $\delta_{C}$, the better balanced the DCP-CWGC becomes, resulting in a more stable detection result for the corresponding combinatorial pooling arrangement. Since a code *C* can be identically represented by its incidence matrix *H*, we also use $W_{H}$ and $\delta_{H}$ to denote the balance vector of *C* and its deviation, respectively.

Clearly, when m≥2r+1, if there exists a DCP-CWGC with maximum length, it must have perfect balance.

Proposition 2. If there exists an $\left(m,r,n=\left(\begin{array}{c} m\\ r \end{array}\right)\right)$ DCP-CWGC C, it must have a perfect balance vector $W_{C}=(w_{1},\ldots ,w_{m})=\left(\left(\begin{array}{c} m-1\\ r-1 \end{array}\right),\ldots ,\left(\begin{array}{c} m-1\\ r-1 \end{array}\right)\right)$.

The construction of perfect balance codes from an arbitrary set of binary vectors with constant weight *r* is equivalent to the *r*-set multi-cover problem, a generalization of the *r*-set cover problem, which is well known to be NP-complete for r≥3 (Garey and Johnson 1979). Likewise, computing a Gray code ordering for an arbitrary set of binary vectors with constant weight *r* is NP-complete (Merino *et al.* 2024) and remains so with the OR-sum constraint. Our considered problem is the combination of the two, but allowing us to choose codewords from the whole set S(m,r); that is, given parameters (m,r,n) as input, the desired output is a Gray code of length *n* from S(m,r) that satisfies the OR-sum constraint and maintains perfect balance. In our setting, the situation is simplified to a degree as we have access to all possible *r* combinations of length *m* when constructing our codes. This makes each problem more tractable when considered individually; however, the combination of these two problems may retain the hardness of the original problems. When combining these problems, we are attempting to compute a Gray code ordering and a set multi-cover simultaneously.

Thus, in the following sections, we will relax the stringent perfect balance requirement, and present two numerical algorithms that construct DCP-CWGCs exhibiting a nearly perfect balance property with flexible parameters.

## 4 Branch-and-bound algorithm

In this section, we propose a branch-and-bound algorithm (BBA) to construct balanced DCP-CWGCs with flexible parameters (m,r,n). BBA proceeds by depth-first heuristic search for a path in an address-union bipartite graph. While traversing this graph, BBA evaluates the balance property of all potential next nodes from the current state and selects the node that best maintains balance. Once the path reaches the target length, the algorithm terminates and returns the DCP-CWGC as the sequence of addresses in the path.

An example of the BBA traversing the bipartite graph is presented in Supplementary Fig. 1. The nodes in the graph are divided into address nodes (*a*, rectangular nodes) and union nodes (*u*, circular nodes). Given the constant weight for addresses and OR-sums of consecutive addresses, the set of all possible addresses *AN* equals to S(m,r), and the set of all possible unions *UN* equals to S(m,r+1). We define Adj(u) as the subset of *AN* incident to union *u* such that bitwise OR-sum of *u* and every address a∈Adj(u) equals *u*:


∀a∈Adj(u):a∨u=u.

Similarly, we define Adj(a) as the subset of *UN* incident to address *a* such that the bitwise OR-sum of *a* and every union u∈Adj(a) equals *u*:


∀u∈Adj(a):a∨u=u.

We denote with *A* the address nodes and *U* the union nodes of the path.

BBA is initiated with an arbitrary address $a_{1}\in AN$ and arbitrary union $u_{1}\in UN$ such that $u_{1}\in \mathrm{Adj}(a_{1})$. Next, a balance-optimized path A∪U containing *n* address nodes is determined heuristically, alternating between address nodes *a* and union nodes *u* and selecting nodes that maintain the balance best.

Here, we outline the specific steps for BBA (Supplementary Algorithm 1), with pseudocode for the core function SearchPath detailed in Supplementary Function 1. Note that sometimes the path search may reach a dead end, that is, for node *p*, Adj(p)\(A∪U) may be an empty set. To avoid dead ends in the graph, the SearchPath function operates recursively (Supplementary Function 1, lines 15 and 32).

1. We initiate the set of the unions by U=ϕ and the set of the addresses *A* by the address $a_{1}$; if $a_{1}$ is not specified, choose a random $a_{1}\in AN$, $A=\{a_{1}\}$. Choose a random $u_{1}\in \mathrm{Adj}(a_{1})$, $U=\{U_{1}\}$.
2. We choose the next address $a_{2}$ (Supplementary Function 1, lines 23-32) to be added to the path. For each $a\in \mathrm{Adj}(u_{1})$, we calculate the balance penalty score associated with adding *a* to the path, equal to the difference of the variances between a balance vector after adding *a* and the current balance vector. Then we sort the addresses in $\mathrm{Adj}(u_{1})$ according to the penalty associated with them. We choose *a* with minimal penalty, that is, the first node in the sorted $\mathrm{Adj}(u_{1})$, add it to *A* as the next address $a_{2}$: $A\leftarrow A\cup \{a_{2}\}=\{a_{1},a_{2}\}$, and continue the search. If a recursive branch reaches a dead end, the algorithm backtracks and replaces $a_{2}$ with the next candidate in the sorted $\mathrm{Adj}(u_{1})$, repeating until a feasible continuation is found.
3. We choose the next union $u_{2}$ (Supplementary Function 1, lines 6-15) to be added to *U*, in analogy to step 2 for finding the next address. We update *U* by adding $u_{2}$: $U\leftarrow U\cup \{u_{2}\}=\{u_{1},u_{2}\}$. If the subsequent path search meets a dead end, $u_{2}$ is replaced with the next candidate in $\mathrm{Adj}(a_{2})$.
4. We proceed further as described in steps 2 and 3, alternating between updating *A* and *U*. We stop when the length of *A* reaches the desired length *n*. Then *A* is returned as the constructed balanced DCP-CWGC.

## 5 Recursive combination approach

In this section, we describe our recursive combination approach. We show that this approach can combine short DCP-CWGCs to construct $\left(m,r,n=\min\{\left(\begin{array}{c} m\\ r \end{array}\right),\left(\begin{array}{c} m\\ r+1 \end{array}\right)\}\right)$ DCP-CWGCs for any positive integer *r* and m≥r+1. We then pair the recursive combination approach with BBA, and show that we can construct approximately balanced DCP-CWGCs with flexible parameters (m,r,n).

### 5.1 Recursive combination of DCP-CWGCs

The recursive combination approach is based on a combination operation that concatenates the incidence matrices of two DCP-CWGCs. These two component codes are in the “augmented” form, whose incidence matrices have one row as an all-one vector 1 or an all-zero vector 0. We first introduce two useful augmentation operators for notational brevity, then demonstrate the details of the approach.

For a binary vector $v={\left(v_{1},v_{2},\ldots ,v_{m}\right)}^{T}$, we denote its “+” augmented vector as $v^{+}={\left(v_{1},v_{2},\ldots ,v_{m},1\right)}^{T}$, and we denote its “-” augmented vector as $v^{-}={\left(v_{1},v_{2},\ldots ,v_{m},0\right)}^{T}$. For an m×n binary matrix $H={\left[a_{1},a_{2},\ldots ,a_{n}\right]}_{m\times n}$, we denote its “+” augmented matrix as $H^{+}:={\left[\begin{array}{cccc} a_{1} & a_{2} & \cdots & a_{n}\\ 1 & 1 & \cdots & 1 \end{array}\right]}_{(m+1)\times n},$ and denote its “-” augmented matrix as $H^{-}:={\left[\begin{array}{cccc} a_{1} & a_{2} & \cdots & a_{n}\\ 0 & 0 & \cdots & 0 \end{array}\right]}_{(m+1)\times n}.$ Moreover, for a DCP-CWGC *C* whose incidence matrix is *H*, we denote $C^{+}$ as the augmented DCP-CWGC whose incidence matrix is $H^{+}$, and denote $C^{-}$ as the augmented DCP-CWGC whose incidence matrix is $H^{-}$. These two operators may be repeatedly applied.

The next lemma gives the basic idea of the recursive combination approach, and the proof is straightforward.

Lemma 1. Let $H_{1}=[a_{1}^{1},\ldots ,a_{n_{1}}^{1}]$ be the incidence matrix of an $\left(m,r-1,n_{1}\right)$ DCP-CWGC $C_{1}$, $H_{2}=[a_{1}^{2},\ldots ,a_{n_{2}}^{2}]$ be the incidence matrix of an $\left(m,r,n_{2}\right)$ DCP-CWGC $C_{2}$. Suppose $a_{n_{1}}^{1}\vee a_{1}^{2}=a_{1}^{2}$, and $a_{n_{1}}^{1}\vee a_{1}^{2}\neq a_{j}^{1}\vee a_{j+1}^{1}$ for all $1\leq j\leq n_{1}-1$, then

(5) $$H:=[H_{1}^{+},H_{2}^{-}]=\left[\begin{array}{cccccc} a_{1}^{1} & \ldots & a_{n_{1}}^{1} & a_{1}^{2} & \ldots & a_{n_{2}}^{2}\\ 1 & \ldots & 1 & 0 & \ldots & 0 \end{array}\right]$$

is the incidence matrix of an $\left(m+1,r,n_{1}+n_{2}\right)$ DCP-CWGC C, which is obtained by the combination of $C_{1}^{+}$ and $C_{2}^{-}$.

We term $C_{1}$ and $C_{2}$ in Lemma 1 the elementary DCP-CWGCs for the combination, and their augmented codes, $C_{1}^{+}$ and $C_{2}^{-}$, serve as component codes. Utilizing the augmented structure, it is straightforward to generalize Lemma 1 to the combination of multiple DCP-CWGCs.

Corollary 1. Let $C=\{C_{m_{0}},C_{m_{0}+1},\ldots ,C_{m}\}$ be a collection of L elementary DCP-CWGCs, $L=m-m_{0}+1$, where $m_{0}<m$ is a pre-specified positive integer, $C_{j}$ is an $\left(j-1,r-1,n_{j}\right)$ DCP-CWGC, $m_{0}+1\leq j\leq m$; and $C_{m_{0}}$ is an $\left(m_{0},r,n_{m_{0}}\right)$ DCP-CWGC. Suppose that the technical conditions for the combination in Lemma 1 are satisfied, then repeated application of Lemma 1 gives the incidence matrix of an $\left(m,r,\sum_{j=m_{0}}^{m}n_{j}\right)$ DCP-CWGC C.

Note that the above combination operation in Lemma 1 exhibits a recursive nature, where the combined code itself may be used as a component DCP-CWGC for a new combination operation. Based on this recursive combination approach, we have the following:

Theorem 1. *There exists an* $\left(m,r,n=\min\{\left(\begin{array}{c} m\\ r \end{array}\right),\left(\begin{array}{c} m\\ r+1 \end{array}\right)\}\right)$  *DCP-CWGC for any positive integers r and* m≥r+1.

Proof. See supplementary material. □

### 5.2 Recursive combination together with BBA

We paired the recursive combination strategy with BBA, which we will henceforth call rcBBA: BBA generates elementary DCP-CWGCs, which are then augmented and combined using the recursive combination approach (Supplementary Algorithm 2). This way, rcBBA can construct balanced DCP-CWGCs with flexible parameters (m,r,n).rcBBA is based on the iterative construction of the full (m,r,n) DCP-CWGC (schematic in Supplementary Fig. 2). Each iteration consists of three main steps.

1. The length of the elementary DCP-CWGC is determined.
2. The elementary DCP-CWGC is generated by BBA and then transformed into a DCP-CWGC component by augmentation and permutation.
3. The DCP-CWGC component is combined into the base code.

Here we provide an overview of the rcBBA core function RecCombine (Supplementary Function 2), which is applied in each iteration. The technical details can also be found in the supplementary material.

First, we will introduce the notation used in this section. We use *j* to serve as an iteration counter, for which rcBBA starts with j=m, decreases *j* by 1 with each iteration, and when $\left(\begin{array}{c} j-1\\ r \end{array}\right)\leq \left(\begin{array}{c} j-1\\ r-1 \end{array}\right)$, that is, j≤2r, rcBBA switches to the final iteration regime. Thus, when $m\leq m_{0}=2r$, rcBBA jumps directly into the final iteration regime and is completed with BBA. The reasons for selecting $m_{0}=2r$ as the termination condition are discussed in Section Computational Complexity and Simulation Results. We use $W_{\mathrm{res}}$ to denote the so-called residual balance vector, which is determined and updated at the beginning of each iteration. We use *W[k]* to denote its *k*-th element. We use $C_{j}$ to denote the elementary DCP-CWGC in the *j*-th iteration, which is of length $n_{j}$ and has a corresponding incidence matrix $H_{j}$. The addresses in $C_{j}$ are denoted as $a_{i}^{j}$, where $i=1,2,\ldots ,n_{j}$. For a binary vector *a*, we use *a[k]* to denote the *k*-th element in *a*. We denote the base incidence matrix as *H* with the *i*-th address $a_{i}^{H}$, where $i=1,2,\ldots ,n_{H}$, and $n_{H}$ is the number of columns in *H*. We denote the augmentation procedure and permutation operation by adding “ $\tilde{}$ ” and “ ^ ” respectively: for example, the augmented DCP-CWGC ${\tilde{C}}_{j}$ and the permuted DCP-CWGC ${\hat{C}}_{j}$, with corresponding incidence matrices ${\tilde{H}}_{j}$ and ${\hat{H}}_{j}$.

rcBBA with parameters (m,r,n) (Supplementary Algorithm 2) starts with the initialization of the iteration counter *j*: j=m. Then the residual balance vector $W_{\mathrm{res}}$ is initialized: $W_{\mathrm{res}}=(w_{1},w_{2},\ldots ,w_{m})$, where $w_{i}$ is calculated using the floored average value $w=\left\lfloor \frac{r\cdot n}{m}\right\rfloor$. If $\frac{r\cdot n}{m}$ is not an integer, then 1 is added to the first r·n−m·w elements:


(6) $$w_{i}=\{\begin{array}{c} w+1,\;1\leq i\leq r\cdot n-m\cdot w\\ w,\;r\cdot n-m\cdot w<i\leq m \end{array}.$$

Also, the residual index set $I_{\mathrm{res}}$ is initialized: $I_{\mathrm{res}}=\{1,2,\ldots ,j\}$, and the base incidence matrix *H* is initialized as an empty matrix. Next, the length of the elementary DCP-CWGC for the *j*-th iteration $n_{j}$ is determined as the *j*-th element of $W_{\mathrm{res}}$: $n_{j}=w_{j}$. The parameters for the next iteration ($H,j,W_{\mathrm{res}},I_{\mathrm{res}},n_{j},m,r,n$) are then entered into the RecCombine function (Supplementary Function 2).

In each iteration of RecCombine, a new DCP-CWGC component is generated. We must ensure the proper joining of this new component with the existing base incidence matrix so that it will satisfy constraints (1, 2), and (3). Consequently, except for the first iteration, we need to search for a suitable binary vector $b_{j}$ which is used to find a permutation map *P* to turn an augmented DCP-CWGC into a DCP-CWGC component. In this procedure, $b_{j}$ will become the first address of the new component DCP-CWGC. We explain in detail how $b_{j}$ is selected using $I_{\mathrm{res}}$ and how *P* is found in the supplementary material. In the first iteration (j=m), $b_{j}$ is not used. We denote the set of vectors $b_{j}$ that satisfy the requirements as $B_{j}$. Note that there may be no binary vector $b_{j}$ satisfying the requirements in some iteration $j=m-1,\ldots ,m_{0}$, thus the above iterative process may reach a dead end. At this point, the algorithm backtracks to the previous iteration, chooses another $b_{j}\in B_{j}$, and uses that $b_{j}$ to find another component DCP-CWGC to replace the current DCP-CWGC.

RecCombine has two regimes: (1) when $j>m_{0}$ (Supplementary Function 2, lines 13–35); and (2) when $j=m_{0}$ (Supplementary Function 2, lines 2–12). In regime 1, the residual balance vector $W_{\mathrm{res}}$ is updated by subtracting the balance vector of the new incidence matrix: $W_{\mathrm{res}}\leftarrow W_{\mathrm{res}}-W_{{\hat{H}}_{j}}$ after each iteration. If RecCombine reaches $j=m_{0}$, it stops iterating through *j* and switches to the final iteration regime. In this regime, an inverse index permutation is applied to $W_{\mathrm{res}}$ to derive the desired balance vector $W_{\mathrm{des}}$, defined as the first $m_{0}$ components of $P^{-1}(W_{\mathrm{res}})$, which is then passed to the BBA. During the path search, the penalty associated with adding a new node to the path is calculated as the variance of the entries in the difference vector between the current balance and $W_{\mathrm{des}}$. The combined DCP-CWGC is approximately balanced if the incidence matrix $H_{m_{0}}$ of the BBA-generated DCP-CWGC in the last iteration has a balance vector that approximates its desired balance vector $W_{\mathrm{des}}$.

Theorem 2. *The deviation of the combined DCP-CWGC C is upper bounded by*
 

$$\delta_{C}\leq 2\cdot \underset{i=1,\ldots ,m_{0}}{\max}\{|W_{H_{m_{0}}}[i]-W_{\mathrm{des}}[i]|\}+2.$$

Proof. See supplementary material. □

## 6 Computational complexity and simulation results

Both BBA and rcBBA are implemented in our open-source software *codePUB* (detailed documentation at https://codepub.readthedocs.io/). We evaluated their performance in terms of computational complexity and runtime, balance of the DCP-CWGCs, and error detection ability.

### 6.1 Computational complexity and empirical runtime

The computational complexity of BBA is mainly driven by the heuristic balance-optimized path search. Consider a balanced (m,r,n) DCP-CWGC constructed by BBA. For simplicity, we assume that the computational complexity when visiting each node (including computing the variances, sorting the variance vector, etc.) is the same “unit” value. Under this assumption, the overall computational complexity of BBA is directly proportional to the number of nodes that are visited during the path search. Thus, the best-case computational complexity of BBA is linear in *n*, that is, Θ(n). In the worst case, the desired path is not found until the last candidate node is checked in every intermediate stage of the path search procedure. Each address *a* has at most m−r choices in Adj(a) for the next union *u*, and each union *u* has at most *r* choices in Adj(u) for the next address *a*. Thus, the worst-case computational complexity for BBA is upper bounded by $O({\left[r(m-r)\right]}^{n})$.

rcBBA has significantly lower worst-case complexity for the path search procedure than full-length construction by BBA owing to the much smallers length of the elementary DCP-CWGCs. Moreover, the auxiliary overhead associated with the recursive combination procedure (such as augmentation, permutation and combination) is relatively small compared to the complexity on generating elementary DCP-CWGCs by BBA. Therefore, rcBBA generally exhibits lower worst-case computational complexity than BBA.

**Figure 4.**
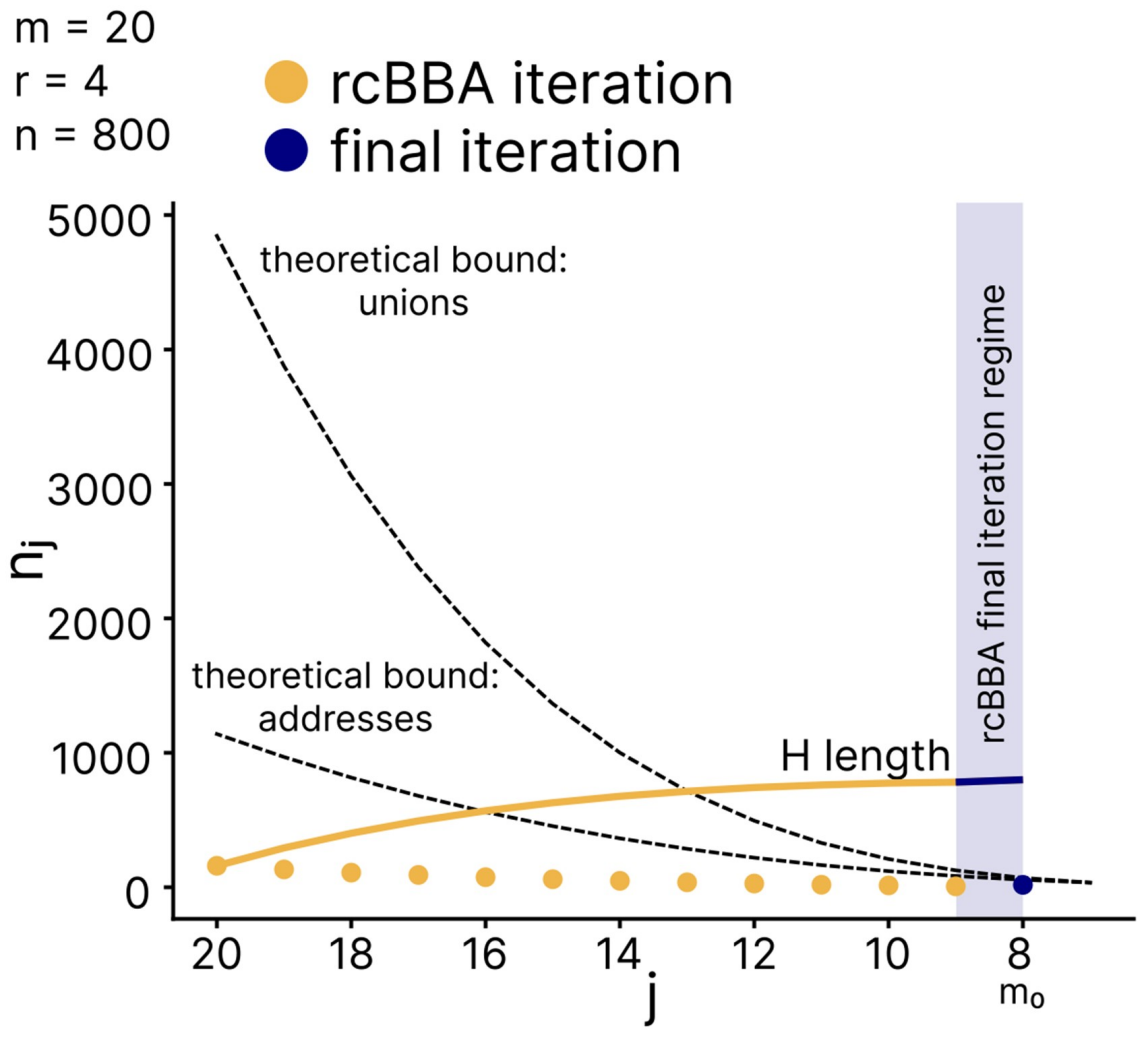
The length of the DCP-CWGC component $n_{j}$ generated with each iteration *j*. Due to rcBBA’s iterative nature with decreasing *j*, the theoretical bound for the length of the component codes, that is, $\text{min}\{\left(\begin{array}{c} j\\ r-1 \end{array}\right),\left(\begin{array}{c} j\\ r \end{array}\right)+1\}$, also decreases with each iteration. To avoid reaching this theoretical bound before construction is completed, rcBBA switches to the final iteration regime at $j=m_{0}=2r$. The orange dots represent elementary codes with length $n_{j}$ produced during each rcBBA iterations, and the blue dot represents the elementary code with length $n_{j}$ produced at the last iteration. The solid line is the cumulative length of the code in the combination iterations.

Of note, with each iteration of rcBBA, the value of *j* decreases, leading to the construction of elementary DCP-CWGCs with progressively smaller *m*. As a result, rcBBA may eventually reach an iteration where the length of the next elementary sequence, $n_{j}$, exceeds the maximum possible number of addresses for given *j* and r−1, specifically when $n_{j}>\left(\begin{array}{c} j\\ r \end{array}\right)$. As this point is approached, the probability of rcBBA failing becomes increasingly high. To prevent this outcome, we included the following stopping criterion: when $\left(\begin{array}{c} j-1\\ r \end{array}\right)\leq \left(\begin{array}{c} j-1\\ r-1 \end{array}\right)$ (Fig. 4), that is, j=2r, rcBBA switches to the final iteration regime.

**Figure 5.**
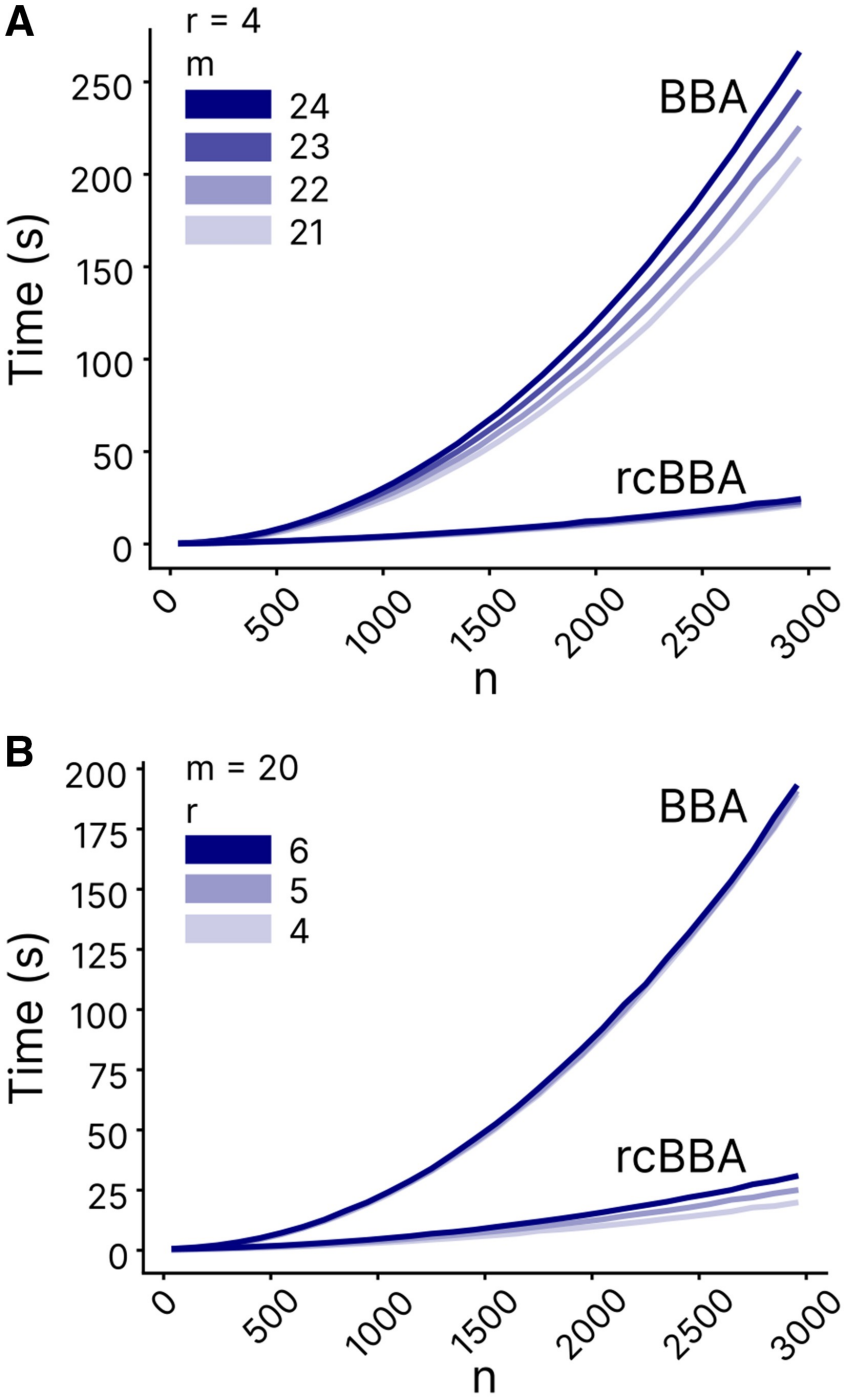
Empirical runtime analyses for BBA and rcBBA. Runtime is evaluated as a function of code length *n*, with varying address lengths *m* (A) and address weights *r* (B). The process was executed on a single core of a general-purpose CPU node equipped with dual Xeon 6252 processors. It utilized 16.5 GB of virtual memory, with 69.4 MB resident in RAM.

We tested the performance of BBA and rcBBA by recording their runtime across a wide range of parameters. Overall, rcBBA demonstrated significantly faster performance than BBA, especially in the regime of large *n* (Fig. 5). The runtime for both algorithms was affected by address length *m* and address weight *r*; as *n* increased, *m* had a greater impact on the runtime than *r* for BBA, while *r* has a greater impact on the runtime than *m* for rcBBA (Fig. 5A versus B). The observed runtimes for both algorithms were much shorter than those estimated from the worst-case computational complexity. However, as *n* approached the theoretical bound $n_{\max}=\text{min}\left\{\left(\begin{array}{c} m\\ r \end{array}\right),\left(\begin{array}{c} m\\ r+1 \end{array}\right)+1\right\}$, the runtimes increased steeply.

**Figure 6.**
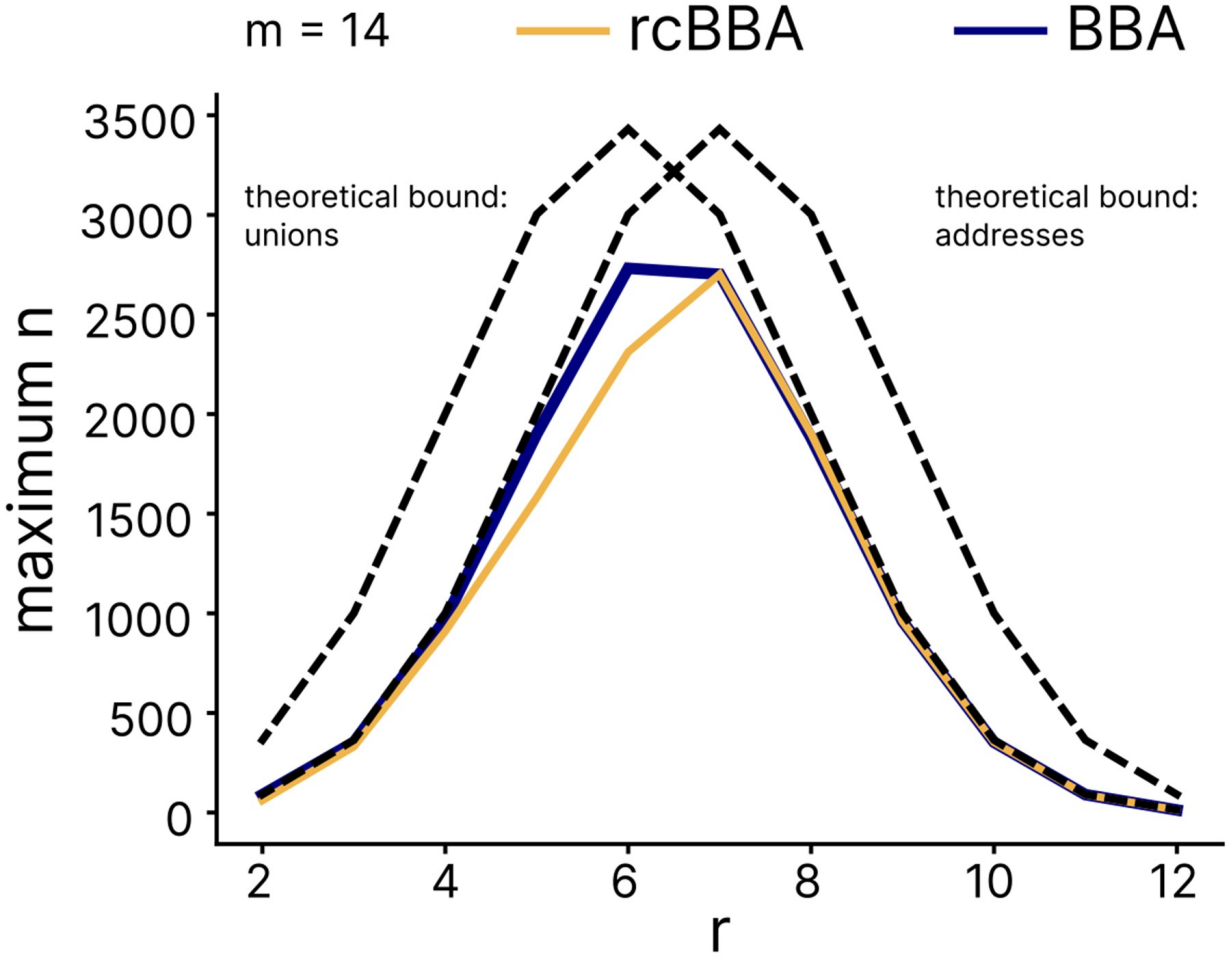
Performance of BBA and rcBBA near the theoretical bound. The theoretical bound lies at the combinatorial coefficient for *n* with $\text{min}\{\left(\begin{array}{c} m\\ r \end{array}\right),\left(\begin{array}{c} m\\ r+1 \end{array}\right)+1\}$. For a fixed number of pools *m*, BBA and rcBBA approach the theoretical bound with increasing address weights *r*.

To evaluate performance near the theoretical bound, we tested both algorithms with values of *n* ranging from $\left\lfloor 0.6\cdot n_{\max}\right\rfloor$ to $\left\lfloor 0.99\cdot n_{\max}\right\rfloor$, covering *m* values from 10 to 15 and *r* values from 2 to 13. If the algorithm returned the code as an empty set or failed to produce a result within the time limit determined by run-time analysis (400 seconds for tested *m* and *r* values), we considered such a test as a failure. For small *r*, BBA slightly outperformed rcBBA, producing a DCP-CWGC with larger *n* for given *m* and *r*, although with a much longer runtime; for r≥7, rcBBA and BBA have the same performance, since rcBBA directly jumps into the final iteration regime which is filled with BBA (Fig. 6).

### 6.2 Balance

**Figure 7.**
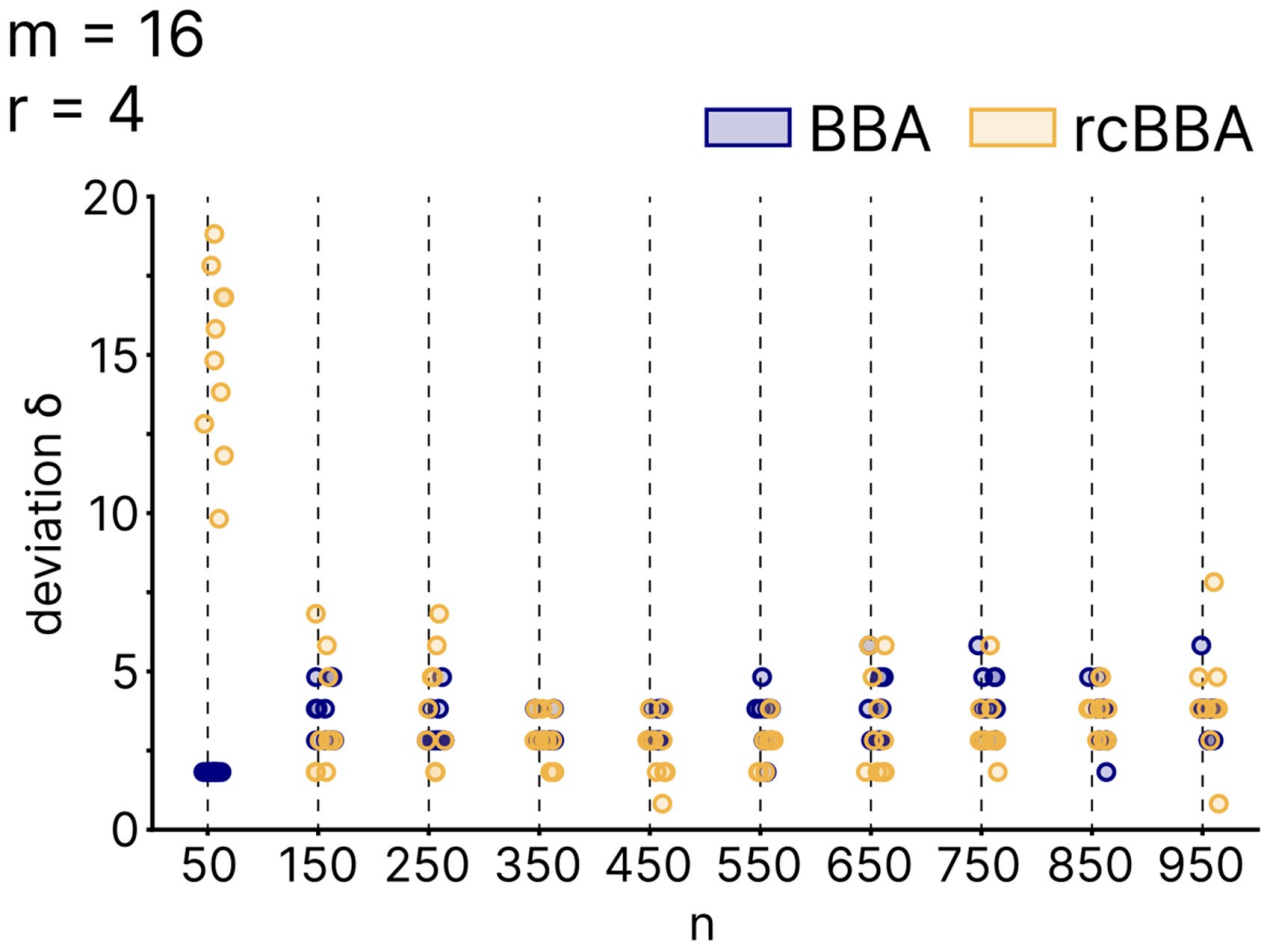
Empirical balance of pooling schemes with DCP-CWGCs. Comparison of the deviations $\delta_{C}=\max(W_{C})-\min(W_{C})$ of BBA-derived and rcBBA-derived experimental balance. Each dot represents one DCP-CWGC with a deviation $\delta_{C}$. A total of 10 runs are shown per parameter set (*m*, *r*, *n*).

We assessed BBA and rcBBA for their ability to generate balanced DCP-CWGCs by measuring the deviation from the perfect balance for different values of *n* (Fig. 7). For very small *n* (e.g. n=50) BBA vastly outperforms rcBBA in generating balanced arrangements. In contrast, both algorithms produced codes with near optimal balance, with deviations not exceeding 8 for *n* ranging from 150 to 950.

### 6.3 Error detection

In a combinatorial pooling experiment with overlapping sequences as items, the number of candidate items is determined by the relative length of the overlap between items and the length of the sequence that generates an experimental signal. If these lengths are identical, then given no experimental errors, the number of target item candidates will be exactly 2. Furthermore, in a combinatorial pooling experiment based on a balanced DCP-CWGC, the number of positive pools remains constant for any positive pair and equals r+1 due to constant-weight (2) and adjacent distance (3) constraints. Consequently, if the number of observed positive pools is not equal to r+1, an experimental error must have occurred, with false positive or false negative results if the number of positive pools is greater or less than r+1, respectively. This deviation from the expected value enables error detection and lets us narrow down the list of potential item candidates based on the positive pools.

**Figure 8.**
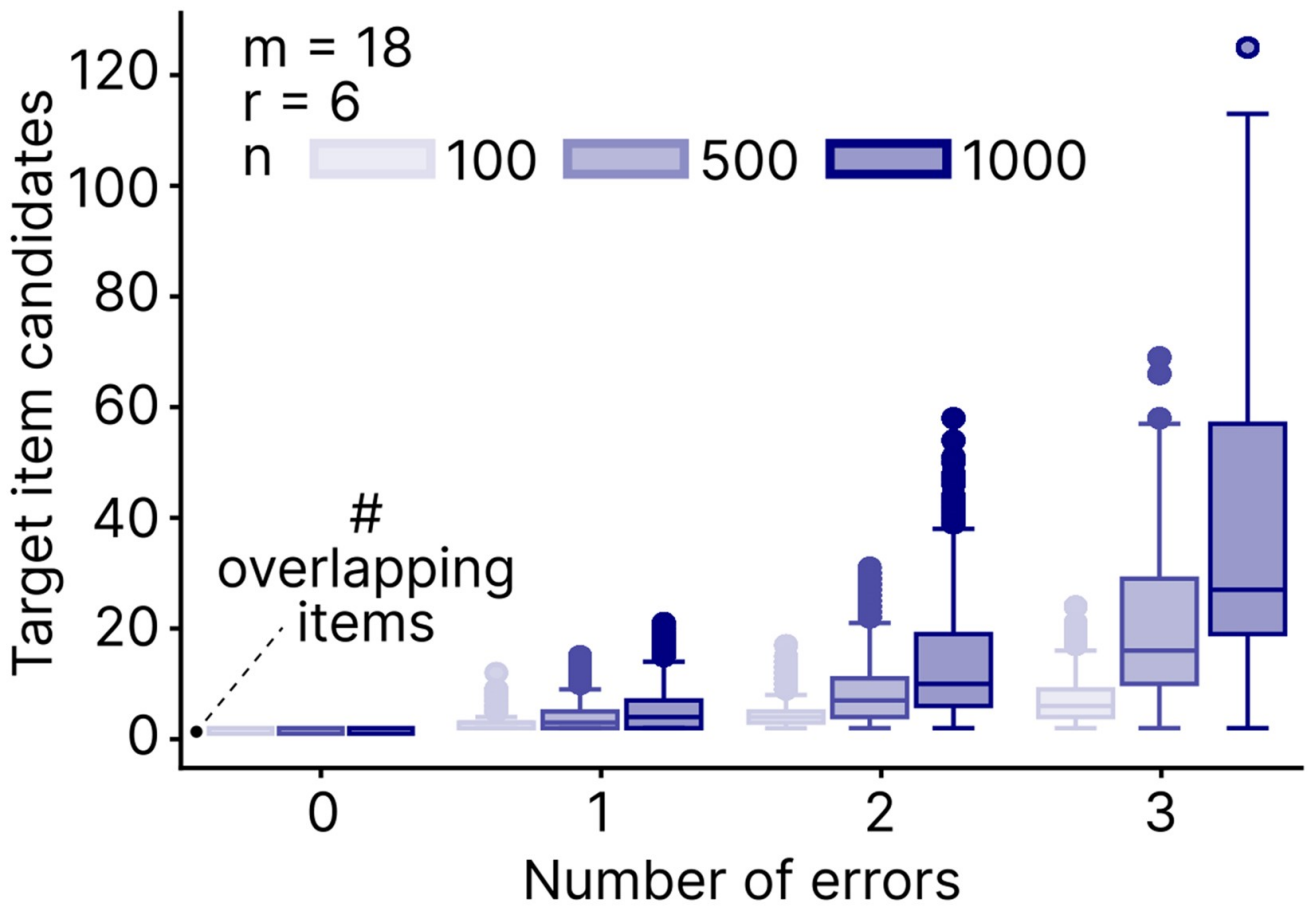
Experimental error detection with DCP-CWGCs-derived pooling schemes. For a fixed number of pools m=18 and address weight r=6, the number of target item candidates depends on the number of tested items *n* and the number of erroneous non-positive pools. Each set of parameters was tested once; for every item, the results of all possible errors were simulated.

We simulated combinatorial pooling experiments using (18,6,n) DCP-CWGCs, where *n* ranged from 100 to 1000, and tested different levels of false-negative experimental errors. We then quantified the number of candidate items expected in these experiments as a function of the number of errors (Fig. 8). Overall, due to the balanced distribution of the DCP-CWGCs, the candidate list remains small (≤5%). For example, with one erroneous non-activated pool, the algorithm can narrow the candidate list to approximately 5 items (for *n *= 100) or up to 30 items (for *n *= 1000).

In summary, BBA alone has a better balance for smaller *n*, with a runtime similar to rcBBA. However, as *n* increases, rcBBA achieves a balance consistent with BBA while maintaining faster performance overall.

## Supplementary Material

btaf611_Supplementary_Data

## Data Availability

The source code is available at GitHub: https://github.com/meyer-lab-cshl/codepub_tests (analyses and figures) and https://github.com/meyer-lab-cshl/codepub (codePUB python package).
