# Supplementary material for: Unbiased and error-detecting combinatorial pooling experiments with balanced constant-weight Gray codes for consecutive positives detection
Source: Bioinformatics. 2025 Nov 13;41(12):btaf611. doi: 10.1093/bioinformatics/btaf611 (PMC12668597; doi:10.1093/bioinformatics/btaf611)
Supplement: btaf611_Supplementary_Data [file btaf611_supplementary_data.pdf]

Supplement to: Unbiased and Error-Detecting Combinatorial Pooling Experiments with Balanced Constant-Weight Gray Codes for Consecutive Positives Detection

Guanchen He†, Vasilisa A. Kovaleva†, Carl Barton, Paul G. Thomas, Mikhail V. Pogorelyy, Hannah V. Meyer*, and Qin Huang*

November 4, 2025

1 Details on BBA

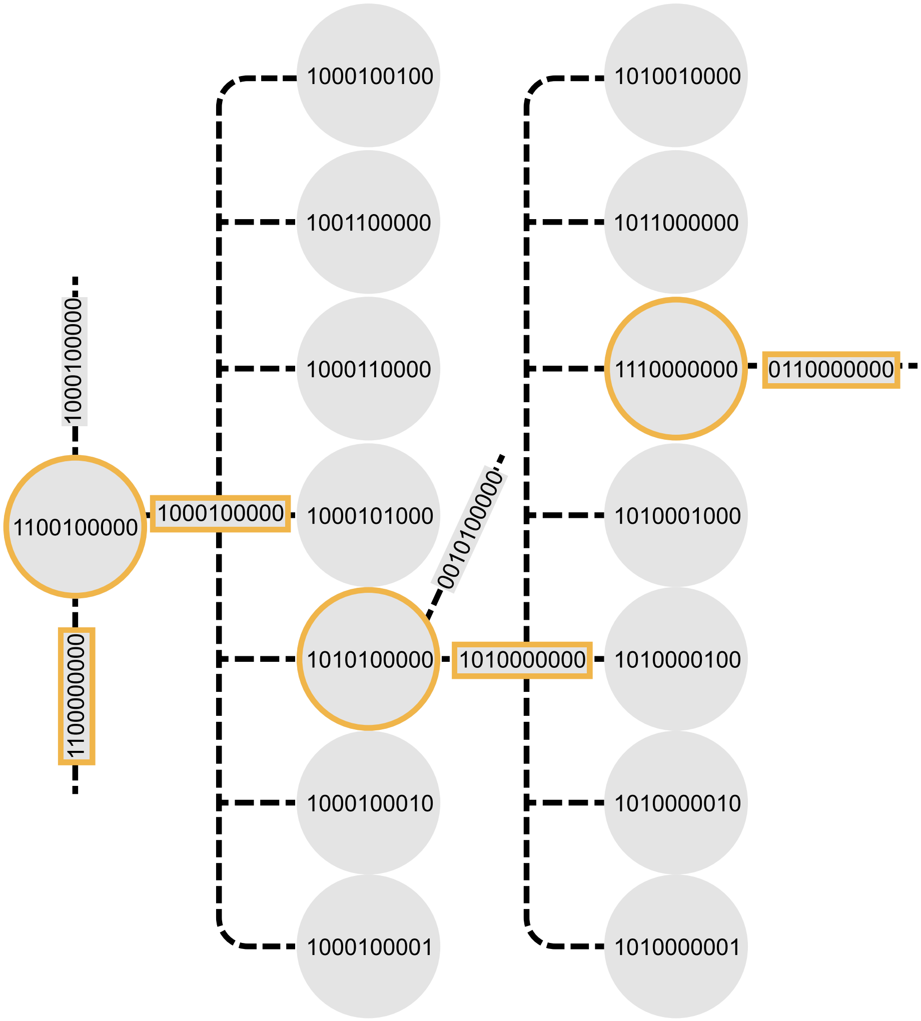

Supplementary Figure 1: An example of the balance-optimized path search in the BBA scheme. Rectangular and circular nodes represent addresses (a) and unions (u), respectively. Yellow nodes indicate the search path taken in which the rectangular ones correspond to a possible segment in an ($m = 10, r = 2, n \geq 4$) DCP-CWGC.

1.1 Pseudocode of BBA

Algorithm 1 Branch-and-bound Algorithm: BBA(m, r, n, a_1, W_{des})

Input: the parameters (m, r, n) of DCP-CWGC, the first address a_1 (optional), the desired balance vector W_{des} (optional).
Output: the constructed balanced DCP-CWGC A.

if a_1 is not specified, choose a random $a_1 \in S(m, r)$.
initiate the path of addresses $A = \{a_1\}$.
initiate path of unions $U = \phi$.
randomly choose current union node u_1 from the subset $Adj(a_1)$.
$A =$ SearchPath(A, U, u_1, W_{des}) if W_{des} is specified; otherwise $A =$ SearchPath(A, U, u_1).
return A.

Function 1 SearchPath(A, U, p, W_{des})

Input: the set of addresses A, the set of unions U, the current node p; the desired balance vector W_{des} (optional).
Output: the found path of addresses A such that $A.length = n$.

if $p \in AN$ and $p \notin A$ **then**
  $A = A \cup \{p\}$.
  if $A.length = n$ **then**
    return A.
  end if
  for $p_{next} \in Adj(p)$ **do**
    if W_{des} is specified **then**
      $penalty_{p_{next}} = variance(W_{des} - W_{A \cup p_{next}})$.
    else
      $penalty_{p_{next}} = variance(W_{A \cup p_{next}}) - variance(W_A)$.
    end if
  end for
  sort $Adj(p)$ using $penalty$ in ascending order $\rightarrow Adj(p)_{sorted}$.
  for $p_{next} \in Adj(p)_{sorted}$ **do**
    $A_{updated} =$ SearchPath$(A, U, p_{next}, W_{des})$ if W_{des} is specified; otherwise $A_{updated} =$ SearchPath(A, U, p_{next}).
    if $A_{updated}.length = n$ **then**
      return $A_{updated}$.
    end if
  end for
  $A = A \backslash \{p\}$.
else if $p \in UN$ and $p \notin U$ **then**
  $U = U \cup \{p\}$.
  for $p_{next} \in Adj(p)$ **do**
    if W_{des} is specified **then**
      $penalty_{p_{next}} = variance(W_{des} - W_{A \cup p_{next}})$.
    else
      $penalty_{p_{next}} = variance(W_{A \cup p_{next}}) - variance(W_A)$.
    end if
  end for
  sort $Adj(p)$ using $penalty$ in ascending order $\rightarrow Adj(p)_{sorted}$.
  for $p_{next} \in Adj(p)_{sorted}$ **do**
    $A_{updated} =$ SearchPath$(A, U, p_{next}, W_{des})$ if W_{des} is specified; otherwise $A_{updated} =$ SearchPath(A, U, p_{next}).
    if $A_{updated}.length = n$ **then**
      return $A_{updated}$.
    end if
  end for
  $U = U \backslash \{p\}$.
end if

2 Details on rcBBA

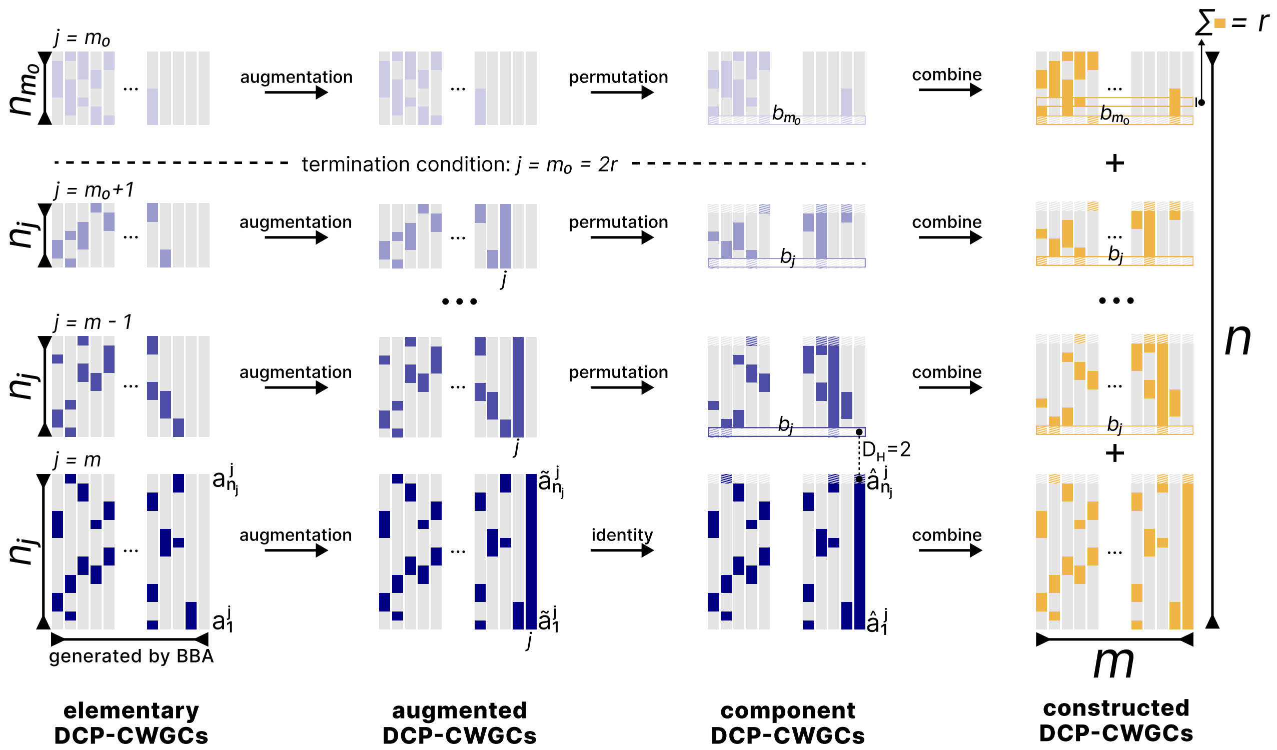

Supplementary Figure 2: The rcBBA scheme. rcBBA is based on iterative construction of the DCP-CWGC with parameters (m, r, n) from elementary DCP-CWGCs. rcBBA iterates with iteration counter j, which starts at $j = m$ and decrements by 1 with each iteration. Each iteration (row) has three main steps (columns): 1) generation of the elementary DCP-CWGC with BBA with parameters $(j-1, r-1, n_j)$, 2) augmentation, and 3) permutation such that the Hamming distance between the first address of the generated DCP-CWGC component b_j and the last address of base code is equal to 2. After all three steps, a component is added to the base code. When rcBBA reaches $j = m_0 = 2r$, it switches to the final iteration regime, where it generates elementary DCP-CCWGC with parameters (m_0, r, n_{m_0}), augments it, permutes the augmented code, and adds the permuted component code to the base code.

2.1 Notational convention

The two operators "+" and "-" can be repeatedly applied. For integers $x, y \geq 0$, we denote "$x+, y-$" as the operation that performs the "+" operator x times and then "$-$" operator y times; and we denote "$x-, y+$" as the operation that performs the "$-$" operator x times and then "+" operator y times. For example, let $v = (v_1, \cdots, v_m)^T$, then $v^{2+,-} = (v_1, \cdots, v_m, 1, 1, 0)^T$; let $H = [a_1, a_2, \cdots, a_n]$, then

$$H^{+,2-} = \begin{bmatrix} a_1 & a_2 & \cdots & a_n \\ 1 & 1 & \cdots & 1 \\ 0 & 0 & \cdots & 0 \\ 0 & 0 & \cdots & 0 \end{bmatrix}.$$

Given the index set $I(a)$ of some binary vector a, we use $I(a)[l]$ to denote the l-th element in $I(a)$, i.e., the index of l-th nonzero element in a.

2.2 The requirements of b_j

Suitable b_j should satisfy the following requirements (1):

- b_j is a binary vector with length m and weight r:

$$b_j \in S(m, r).$$

- each element in the index form of b_j is in the residual index set I_{res}:

$$\forall i \in I(b_j) : i \in I_{res}.$$

- the Hamming distance between b_j and the last address $a^H_{n_H}$ in H equals to 2, i.e. $b_j \in Adj(Adj(a^H_{n_H}))$:

$$D_H(b_j, a^H_{n_H}) = 2.$$

- the union formed by $a^H_{n_H}$ and b_j is distinct from consecutive unions in H:

$$\forall k = 1, 2, ..., n_H - 1 : a^H_{n_H} \vee b_j \neq a^H_k \vee a^H_{k+1}.$$

2.3 Two regimes for rcBBA

2.3.1 if $j > m_0$

- generate a $(j-1, r-1, n_j)$ elementary DCP-CWGC C_j by BBA with the incidence matrix H_j:

$$H_j = [a^j_1, \cdots, a^j_{n_j}].$$

- apply augmentation on H_j:

$$\tilde{H}_j = [\tilde{a}^j_1, \cdots, \tilde{a}^j_{n_j}] := H_j^{+,(m-j)-}.$$

- find a permutation map P on $\{1, 2, \cdots, m\}$, such that (2):
  - if it is in the first iteration ($j = m$), the permutation map is an identity map;
  - P maps elements in $\{1, \cdots, j\}$ one-to-one into I_{res}:

    $$P(\{1, \cdots, j\}) = I_{res};$$

  - the first address of $\tilde{H}_j$ is permuted to b_j:

    $$\tilde{a}^j_1 \to b_j;$$

  - the r-th element of $I(\tilde{a}^j_1)$ is permuted to the r-th element of $I(b_j)$:

    $$I(\tilde{a}^j_1)[r] \to I(b_j)[r].$$

- apply P as a row permutation on $\tilde{H}_j$:

$$\hat{H}_j = [\hat{a}^j_1, \cdots, \hat{a}^j_{n_j}] = P(\tilde{H}_j).$$

- add $\hat{H}_j$ to the base incidence matrix:

$$H \leftarrow [H, \hat{H}_j].$$

- update parameters to prepare for the next iteration: remove the r-th element of $I(b_j)$ from the residual index set I_{res}: $I_{res} \leftarrow I_{res} \backslash \{I(b_j)[r]\}$, update residual balance vector W_{res} by subtracting the balance vector of the new incidence matrix: $W_{res} \leftarrow W_{res} - W_{\hat{H}_j}$, update $j = j - 1$.

- find the new suitable $b_j \in B_j$ that satisfies (1).

- for each b_j, find corresponding n_j equal to the $I(b_j)[r]$-th element of W_{res}, where $I(b_j)[r]$ is the r-th element of the index form of b_j:

$$n_j = W_{res}[I(b_j)[r]].$$

  Sort B_j based on the n_j in descending order.

- for each b_j in the sorted B_j, continue with new parameters: $(H, j, W_{res}, I_{res}, b_j, n_j, m, r, n)$.

2.3.2 if $j = m_0$

- determine the length of the last component DCP-CWGC $\hat{C}_{m_0}$ with incidence matrix $\hat{H}_{m_0}$ by subtracting number of columns of H from the desired length n:

$$n_{m_0} = n - n_H.$$

- randomly select a first address $a_1^{m_0} \in S(m_0, r)$.

- find a permutation map P on $\{1, \cdots, m\}$, such that (3):

  - if $j = m$, permutation map is an identity map;
  - the set $\{1, \cdots, m_0\}$ is mapped one-to-one to I_{res}:

$$P(\{1, \cdots, m_0\}) = I_{res};$$

  - the augmented first address $(a_1^{m_0})^{(m-m_0)-}$ is permuted to b_j:

$$(a_1^{m_0})^{(m-m_0)-} \to b_j.$$

- apply inverse index permutation map P^{-1} on the residual balance vector W_{res}, and determine the desired balance vector W_{des} for BBA in this iteration:

$$W_{des} = \left(P^{-1}(W_{res})[1], \cdots, P^{-1}(W_{res})[m_0]\right).$$

- generate by BBA an (m_0, r, n_{m_0}) elementary DCP-CWGC C_{m_0} with desired balance W_{des}, whose incidence matrix H_{m_0}:

$$H_{m_0} = [a_1^{m_0}, \cdots, a_{n_{m_0}}^{m_0}].$$

- apply augmentation on H_{m_0}:

$$\tilde{H}_{m_0} = [\tilde{a}_1^{m_0}, \cdots, \tilde{a}_{n_{m_0}}^{m_0}] := H_{m_0}^{(m-m_0)-}.$$

- apply P as a row permutation on $\tilde{H}_{m_0}$:

$$\hat{H}_{m_0} = [\hat{a}_1^{m_0}, \cdots, \hat{a}_{n_{m_0}}^{m_0}] = P(\tilde{H}_{m_0}).$$

- update the base incidence matrix $H \leftarrow [H, \hat{H}_{m_0}]$, and return H as the incidence matrix of the constructed DCP-CWGC C.

2.4 Pseudocode of rcBBA

Algorithm 2 Recursive Combination together with BBA: $\texttt{rcBBA}(m, r, n)$

Input: the desired parameters (m, r, n) of DCP-CWGC.
Output: the incidence matrix H of desired DCP-CWGC.

initialize the base incidence matrix H as an empty matrix.
initialize iteration counter $j = m$.
initialize the residual balance vector $W_{res} = (w_1, w_2, \ldots, w_m)$ by: $w_i = w + 1$ if $1 \le i \le r \cdot n - m \cdot w$; $w_i = w$ if $r \cdot n - m \cdot w < i \le m$, where $w = \lfloor \frac{r \cdot n}{m} \rfloor$.
initialize the residual index set $I_{res} = \{1, 2, ..., j\}$, and let $n_j = w_j$.
$H = \texttt{RecCombine}(H, j, W_{res}, I_{res}, n_j, m, r, n)$.
return H.

Function 2 $\texttt{RecCombine}(H, j, W_{res}, I_{res}, b_j, n_j, m, r, n)$

Input: the base incidence matrix H, the iteration counter j, the residual balance vector W_{res}, the residual index set I_{res}, the first binary address for the next DCP-CWGC b_j (optional), the length of the next DCP-CWGC n_j, the number of pools m, the weight of binary address r, and the desired code length n.
Output: the incidence matrix H of the constructed DCP-CWGC.

termination condition: $m_0 = 2r$.
if $j = m_0$ **then**
  $n_{m_0} = n - n_H$.
  randomly select $a_1^{m_0} \in S(m_0, r)$.
  find a permutation map P on $\{1, \cdots, m\}$ such that it satisfies (3).
  permute W_{res} with P^{-1}, and let $W_{des} = \big(P^{-1}(W_{res})[1], \cdots, P^{-1}(W_{res})[m_0]\big)$.
  $C_{m_0} = \texttt{BBA}(m_0, r, n_{m_0}, a_1^{m_0}, W_{des})$. Its incidence matrix: H_{m_0}.
  if $H_{m_0} \neq \phi$ **then**
    augmentation: $\tilde{H}_{m_0} = H_{m_0}^{(m-m_0)-}$.
    row permutation: $\hat{H}_{m_0} = P(\tilde{H}_{m_0})$.
    return $[H, \hat{H}_{m_0}]$.
  end if
else if $j > m_0$ **then**
  if $n_H = n$ **then**
    return H.
  end if
  $C_j = \texttt{BBA}(j-1, r-1, n_j)$. Its incidence matrix: H_j.
  if $H_j \neq \phi$ **then**
    augmentation: $\tilde{H}_j = H_j^{+,(m-j)-}$.
    find a permutation map P on $\{1, \cdots, m\}$, such that it satisfies (2).
    row permutation: $\hat{H}_j = P(\tilde{H}_j)$.
    update base incidence matrix: $H = [H, \hat{H}_j]$.
    update W_{res}: $W_{res} = W_{res} - W_{\hat{H}_j}$, and update $I_{res} = I_{res} \backslash \{I(b_j)[r]\}$.
    update j: $j = j - 1$.
    search the set B_j consisting of binary vectors b_j's that satisfy (1).
    if $B_j \neq \phi$ **then**
      for each b_j, find corresponding $n_j = W_{res}[I(b_j)[r]]$ and sort b_j according to their n_j in the descending order.
      for b_j in sorted B_j **do**
        $H = \texttt{RecCombine}(H, j, W_{res}, I_{res}, b_j, n_j, m, r, n)$.
        if $n_H = n$ **then**
          return H.
        end if
      end for
    end if
  end if
end if

3 Supplementary Proofs

3.1 Proof of Theorem 1

The proof utilizes a useful observation that the set of incidence matrices of codes in DCP-CWGC(m, r, n) is invariant under row permutations.

Lemma 1 *Let H be an incidence matrix of an (m, r, n) DCP-CWGC, then for any $m \times m$ permutation matrix P, $P \cdot H$ is also an incidence matrix of an (m, r, n) DCP-CWGC.*

Given r fixed, one of the starting codes for the combination is chosen as a maximal strictly balanced $\left(2r+1, r, \binom{2r+1}{r}\right)$ DCP-CWGC. Such a code is proven to exist from the well-known *middle two levels problem* [1,2]. It is straightforward to verify that the solution to the middle two levels problem satisfies the constraints of DCP-CWGCs. Therefore, any solution to the middle two levels problems C is a $\left(2r+1, r, n = \binom{2r+1}{r}\right)$ DCP-CWGC and we get the following lemma.

Lemma 2 *There exists a $\left(2r+1, r, n = \binom{2r+1}{r}\right)$ DCP-CWGC for any positive integer r.*

Following on from the previous lemma, we first establish the existence of DCP-CWCGs for any positive integers r and $m \geq 2r+1$. The proof is by induction on r.

For $r = 1$, it is straightforward that there exists an $(m, 1, m)$ DCP-CWGC for any $m \geq 2r+1 = 3$.

For any fixed integer $r \geq 2$, suppose that there exist $\left(m, r-1, \binom{m}{r-1}\right)$ DCP-CWGCs for all $m \geq 2(r-1)+1$, and we denote their incidence matrices as $H_{m,r-1,\binom{m}{r-1}} = [a_1^{(m,r-1)}, \cdots, a_{\binom{m}{r-1}}^{(m,r-1)}]$, respectively. Moreover, assume that for all $m \geq 2r+1$, there exists a column vector $y_{m,r-1}$ of Hamming weight r for $H_{m,r-1,\binom{m}{r-1}}$, such that $y_{m,r-1} \vee a_{\binom{m}{r-1}}^{(m,r-1)} = y_{m,r-1}$ and $y_{m,r-1} \neq a_l^{(m,r-1)} \vee a_{l+1}^{(m,r-1)}$, $\forall 1 \leq l \leq \binom{m}{r-1} - 1$. This column vector enables the application of the recursive combination approach.

Based on these DCP-CWGCs in DCP-CWGC$(m, r-1)$, we will construct an $\left(m, r, \binom{m}{r}\right)$ DCP-CWGC with incidence matrix $H_{m,r,\binom{m}{r}} = [a_1^{(m,r)}, \cdots, a_{\binom{m}{r}}^{(m,r)}]$ for any $m \geq 2r+1$. Moreover, we will also prove that $\forall m \geq 2r+1$, there exists a column vector $y_{m,r}$ of Hamming weight $r+1$ for $H_{m,r,\binom{m}{r}}$, such that $y_{m,r} \vee a_{\binom{m}{r}}^{(m,r)} = y_{m,r}$ and $y_{m,r} \neq a_l^{(m,r)} \vee a_{l+1}^{(m,r)}$, $\forall 1 \leq l \leq \binom{m}{r} - 1$.

The construction is by induction on m. By Lemma 2, there exists a $\left(2r+1, r, \binom{2r+1}{r}\right)$ DCP-CWGC, and we denote its incidence matrix as $H_{2r+1,r,\binom{2r+1}{r}} = [a_1^{(2r+1,r)}, \cdots, a_{\binom{2r+1}{r}}^{(2r+1,r)}]$. Since the construction in Lemma 2 comes from the middle two levels problem, there exists a column vector $y_{2r+1,r}$ of Hamming weight $r+1$, such that $y_{2r+1,r} \vee a_{\binom{2r+1}{r}}^{(2r+1,r)} = y_{2r+1,r}$ and $y_{2r+1,r} \neq a_l^{(2r+1,r)} \vee a_{l+1}^{(2r+1,r)}$, for all $1 \leq l \leq \binom{2r+1}{r} - 1$.

Suppose that we have constructed an $\left(m, r, \binom{m}{r}\right)$ DCP-CWGC for some $m \geq 2r+1$, whose $m \times \binom{m}{r}$ incidence matrix is $H_{m,r,\binom{m}{r}} = [a_1^{(m,r)}, a_2^{(m,r)}, \cdots, a_{\binom{m}{r}}^{(m,r)}]$, and there exists a corresponding column vector $y_{m,r}$ of Hamming weight $r+1$ satisfying $y_{m,r} \vee a_{\binom{m}{r}}^{(m,r)} = y_{m,r}$ and $y_{m,r} \neq a_l^{(m,r)} \vee a_{l+1}^{(m,r)}$, for all $1 \leq l \leq \binom{m}{r} - 1$. From the induction hypothesis on r, there exists an $\left(m, r-1, \binom{m}{r-1}\right)$ DCP-CWGC, whose incidence matrix is denoted as $H_{m,r-1,\binom{m}{r-1}}$, and there exists a corresponding column vector $y_{m,r-1}$. Thus, we may apply a suitable row permutation P on $H_{m,r,\binom{m}{r}}$, giving us $H'_{m,r,\binom{m}{r}} = P \cdot H_{m,r,\binom{m}{r}}$, such that $P \cdot a_1^{(m,r)} = y_{m,r-1}$. Then, by the combination of two augmented DCP-CWGCs, we know that the following matrix

$$H_{m+1,r,\binom{m+1}{r}} = [a_1^{(m+1,r)} \cdots, a_{\binom{m+1}{r}}^{(m+1,r)}] := \begin{bmatrix} H_{m,r-1,\binom{m}{r-1}} & H'_{m,r,\binom{m}{r}} \\ \mathbf{1} & \mathbf{0} \end{bmatrix}$$

gives the incidence matrix of an $\left(m+1, r, \binom{m+1}{r}\right)$ DCP-CWGC. Moreover, the column vector $y_{m+1,r} = (P \cdot y_{m,r})^-$ has Hamming weight $r+1$ and satisfies $y_{m+1,r} \vee a_{\binom{m+1}{r}}^{(m+1,r)} = y_{m+1,r}$ and $y_{m+1,r} \neq a_l^{(m+1,r)} \vee a_{l+1}^{(m+1,r)}$, for all $1 \leq l \leq \binom{m+1}{r} - 1$.

As a result, for any $r \geq 1$ and any $m \geq 2r+1$, we have constructed an $\left(m, r, \binom{m}{r}\right)$ DCP-CWGC.

Then, we prove that for any positive integers r and any $m \geq r+1$, there exists an $(m, r, n = \min\{\binom{m}{r}, \binom{m}{r+1}\})$ DCP-CWCG.

The proof utilizes an observation that a path between $S(m,r)$ and $S(m,r+1)$ is isomorphic to a path between $S(m,m-r)$ and $S(m,m-r-1)$. From the previous discussion, we know that there exists an $\left(m, r, \binom{m}{r}\right)$ DCP-CWGC C whose incidence matrix is $H_{m,r,\binom{m}{r}} = [a_1^{(m,r)}, \cdots, a_{\binom{m}{r}}^{(m,r)}]$ for any $m \geq 2r+1$. Moreover, there exists a column vector $y_{m,r}$ of weight $r+1$, such that $y_{m,r} \vee a_{\binom{m}{r}}^{(m,r)} = y_{m,r}$ and $y_{m,r} \neq a_l^{(m,r)} \vee a_{l+1}^{(m,r)}$ for all $1 \leq l \leq \binom{m}{r} - 1$. Thus, we may establish a path of length $2 \cdot \binom{m}{r}$ between $S(m,r)$ and $S(m,r+1)$, which contains $\binom{m}{r}$ nodes in $S(m,r)$ and $\binom{m}{r}$ nodes in $S(m,r+1)$. By flipping all the "1"s to "0"s and "0"s to "1"s in all binary vectors in the path, it gives a path of length $2 \cdot \binom{m}{r}$ between $S(m,m-r)$ and $S(m,m-r-1)$, which contains $\binom{m}{m-r}$ nodes in $S(m,m-r)$ and $\binom{m}{m-r}$ nodes in $S(m,m-r-1)$. This path is identical to an $(m, r' = m-r-1, n = \binom{m}{r'+1})$ DCP-CWGC $\bar{C}$. Given m fixed, the $(m, r, \binom{m}{r})$ DCP-CWGC C has parameter r in the range $1 \leq r \leq \lfloor \frac{m-1}{2} \rfloor$. Thus, $r' = m-r-1$ is in the range $\lceil \frac{m-1}{2} \rceil \leq r' \leq m-2$. This leads to the existence of an $(m, r', \binom{m}{r'+1})$ DCP-CWGC $\bar{C}$ for any m, r' such that $m-2 \geq r' \geq \lfloor \frac{m-1}{2} \rfloor$. Moreover, for $r' = m-1$, it is trivial that there exists an $(m, r', \binom{m}{r'+1})$ DCP-CWGC. As a result, there exists $(m, r, n = \min\{\binom{m}{r}, \binom{m}{r+1}\})$ DCP-CWCGs for any positive integers r and $m \geq r+1$, and the theorem holds.

3.2 Proof of Theorem 2

Due to the augmented structure of component DCP-CWGCs and the fact that their lengths are recursively determined in each iteration, we have the following identities:

$$\begin{cases} n_j = W_{res}^m[I(b_j)[r]] - \sum_{j'=j+1}^{m} W_{\hat{H}_{j'}}[I(b_j)[r]], & \forall j = m, \cdots, m_0+1; \\ n_{m_0} = n - \sum_{j=m_0+1}^{n} n_j; \end{cases} \tag{4}$$

where W_{res}^m is the initialized residual balance vector (in Supplementary Algorithm 2, line 3). In the main text, it is denoted simply as W_{res}; however, since iteration-specific notation is required for this proof, we use W_{res}^m, representing the residual balance vector at iteration m. Note that W_{res}^m has a deviation at most 1. Since the components of W_{res}^m take value from $\{w, w+1\}$ and the lengths of the component DCP-CWGCs are determined by (4), $w = \lfloor \frac{r \cdot n}{m} \rfloor$, it is straightforward that $W_H[I(b_m)[r]], \cdots, W_H[I(b_{m_0+1})[r]] \in \{w, w+1\}$. Denote

$$I_{res}^{m_0} = \{1, \cdots, m\} \backslash \{I(b_m)[r], \cdots, I(b_{m_0+1})[r]\}.$$

Moreover, let us denote $W_{res}^{m_0}$ to be the residual balance vector at the m_0-th iteration, and denote P to be the permutation map in the m_0-th iteration. Then, we have

$$\begin{aligned} \delta_C &= \max_{i=1,\cdots,m} \{W_H[i]\} - \min_{i=1,\cdots,m} \{W_H[i]\} \\ &\leq \max_{i \in I_{res}^{m_0}} \{W_H[i]\} - \min_{i \in I_{res}^{m_0}} \{W_H[i]\} + 1 \\ &= \max_{i \in I_{res}^{m_0}} \left\{ W_{\hat{H}_{m_0}}[i] + \sum_{j=m_0+1}^{m} W_{\hat{H}_j}[i] \right\} \\ &\qquad - \min_{i \in I_{res}^{m_0}} \left\{ W_{\hat{H}_{m_0}}[i] + \sum_{j=m_0+1}^{m} W_{\hat{H}_j}[i] \right\} + 1 \\ &= \max_{i \in I_{res}^{m_0}} \left\{ W_{\hat{H}_{m_0}}[i] + W_{res}^m[i] - W_{res}^{m_0}[i] \right\} \\ &\qquad - \min_{i \in I_{res}^{m_0}} \left\{ W_{\hat{H}_{m_0}}[i] + W_{res}^m[i] - W_{res}^{m_0}[i] \right\} + 1 \\ &\leq \max_{i \in I_{res}^{m_0}} \left\{ W_{\hat{H}_{m_0}}[i] - W_{res}^{m_0}[i] \right\} + \max_{i \in I_{res}^{m_0}} \{W_{res}^m[i]\} \\ &\quad - \min_{i \in I_{res}^{m_0}} \left\{ W_{\hat{H}_{m_0}}[i] - W_{res}^{m_0}[i] \right\} - \min_{i \in I_{res}^{m_0}} \{W_{res}^m[i]\} + 1 \\ &\leq 2 \cdot \max_{i \in I_{res}^{m_0}} \left\{ |W_{\hat{H}_{m_0}}[i] - W_{res}^{m_0}[i]| \right\} + 2 \\ &= 2 \cdot \max_{i \in P^{-1}(I_{res}^{m_0})} \left\{ |W_{H_{m_0}}[i] - W_{des}[i]| \right\} + 2 \\ &= 2 \cdot \max_{i=1,\cdots,m_0} \left\{ |W_{H_{m_0}}[i] - W_{des}[i]| \right\} + 2. \end{aligned}$$

References

[1] T. Mütze, "Proof of the middle levels conjecture," *Proceedings of the London Mathematical Society*, vol. 112, no. 4, pp. 677–713, 2016.

[2] ——, "A book proof of the middle levels theorem," *Combinatorica*, vol. 44, no. 1, pp. 205–208, 2024.
